# 15 year experience of endoscopic atraumatic coronary artery bypass graft (EndoACAB): a single centre experience of a tertiary referral centre

**DOI:** 10.1186/1749-8090-10-S1-A10

**Published:** 2015-12-16

**Authors:** Alan Soo, Richard Trimlett, Rashmi Yadav, Anthony de Souza

**Affiliations:** 1 Department of Cardiac Surgery, Royal Brompton Hospital, London, UK

## Background/Introduction

EndoACAB is a relatively new procedure and remains uncommon, practiced only in specialist hospitals. This is mostly due to requirement of specialised instruments and lengthy learning curve. In this procedure, the left internal mammary artery (LIMA) is harvested endoscopically and anastomosed to the left anterior descending (LAD) artery off pump through a small left micro-thoracotomy incision. There is a wide variation of the way this procedure is performed.

## Aims/Objectives

In this study, we described our experience of performing this procedure over the last 15 years.

## Method

The hospital cardiac surgery database (PATS) was retrospectively reviewed. Patient characteristics and outcome including mortality and post-operative complication are reported.

## Results

A total of 475 patients underwent this procedure from January 2000 to May 2015. Of these patients, 420 (88.4.9%) underwent the procedure on an elective basis, 52 (10.9%) on an urgent basis and 3 (0.63%) as emergency.

There were 2 (0.46%) mortalities and 1 (0.2%) requiring reoperation for bleeding. Other documented complications include pleural and pericardial effusion requiring drainage, atrial and ventricular arrhythmias and permanent pacemaker insertion. There was 37 (7.8%) conversion to sternotomy and convention on pump CABG with the commonest reason being damage to IMA and severe adhesions in the left pleural cavity.

## Discussion/Conclusion

Despite its technical challenge, EndoACAB can be performed reproducibly with excellent outcome. We advocate that this procedure should be offered to all suitable patients (single vessel LAD stenosis or multivessel coronary artery disease whereby the non-LAD coronaries are amenable to percutaneous intervention).

